# Complications of CT-guided transthoracic lung biopsy: A short report on current literature and a case of systemic air embolism

**DOI:** 10.1007/s00508-018-1317-0

**Published:** 2018-01-23

**Authors:** David Lang, Viktoria Reinelt, Andreas Horner, Kaveh Akbari, Franz Fellner, Petra Lichtenberger, Bernd Lamprecht

**Affiliations:** 1 Department of Pulmonology, Kepler University Hospital, Med Campus III, Krankenhausstraße 9, 4020 Linz, Austria; 2 Central Radiology Institute, Kepler University Hospital, Linz, Austria

**Keywords:** Air embolism, Percutaneous CT-guided transthoracic biopsy, Lung biopsy, Lung cancer, Pulmonary nodule

## Abstract

Percutaneous computed tomography (CT)-guided transthoracic needle biopsy (PCNB) is a common diagnostic procedure and is especially indispensable in thoracic oncology. Complications, such as pulmonary hemorrhage and pneumothorax are frequent, but usually easy to manage. Systemic air embolism is a rare but relevant adverse event and its true incidence is probably underestimated, as not all cases may become clinically apparent. We present a case of systemic air embolism following a core-needle biopsy of a left upper lobe lesion, where immediately after the procedure CT scans documented air in the thoracic aorta and in the left ventricle. In this context, we review the current literature on technical aspects as well as on frequent and infrequent major complications of PCNB, together with risk factors, emergency treatment and prevention strategies.

## Introduction

Percutaneous CT-guided transthoracic needle biopsy (PCNB) is a common procedure in the diagnostic work-up of pulmonary lesions difficult to reach by bronchoscopy. Indications include the determination of malignancy in primary lesions, the acquisition of samples for further analyses guiding treatment decisions, staging in case of suspected metastases and the assessment of microbiology in suspected infections [1–3]. Pneumothorax and pulmonary hemorrhage are by far the most frequent complications following PCNB. Although overall procedural complication rates up to 45% have been reported, further interventions, such as chest tube placement are required in less than 10% [3–5]. Other adverse events, such as systemic air embolism, needle tract seeding, and death have a much lower incidence, occurring in less than 1% of cases [3, 4].

In this article we present and discuss a well-documented case of systemic air embolism and give a brief overview of frequent and rare complications following PCNB.

## Case report

### Patient history

The patient was a 69-year-old man who had primarily undergone lobectomy of the left upper lobe and partial pulmonary artery resection for a pulmonary adenocarcinoma in August 2012. According to the 7th lung cancer TNM classification and staging system, the tumor was described as pT4 N1 G3 L1 V1 Pn0, resulting in a stage IIIA. Consequently, the patient underwent 4 cycles of an adjuvant chemotherapy with carboplatin and pemetrexed until November 2012, followed by an observational period. In September 2013, new symptomatic cerebral metastases were treated by whole brain radiotherapy, whereas no other signs of other tumor activity were present. After a stable disease period until June 2015, routine controls suggested thoracic progression with suspicious peripheral nodules in both lower lobes. Retrieval of specimens by bronchoscopy was unsuccessful, but tissue was required for molecular pathology and potential target therapy that had subsequently become available.

### CT-guided core needle biopsy and systemic air embolism

**Fig. 1:**
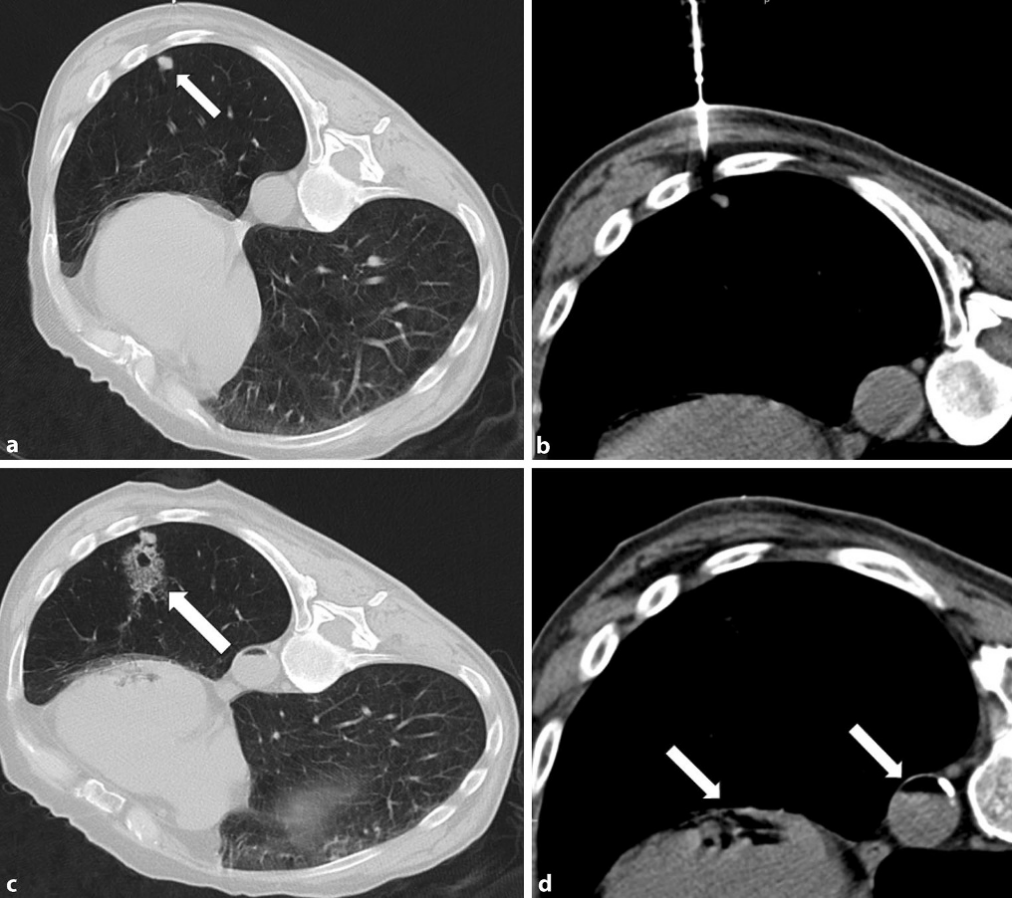
Localization of the lesion in the left lower lobe (**a**) and CT imaging during the intervention, **b** with the patient placed in prone position. The control CT scan immediately after the biopsy shows parenchymal hemorrhage (**c**) and an air level in the thoracic aorta and the left ventricle (**c**,**d**)

The treating specialists referred the patient to interventional radiology for a PCNB to be performed. The patient was positioned in a prone to right-lateral manner (Fig. 1a). During the procedure, using an 18 G Manan™ (Manan Medical Products, Inc., Wheeling, IL, United States) core biopsy device, the target lesion could be optimally reached and several samples were taken (Fig. 1b). Immediately after, the routine control CT scan showed parenchymal hemorrhage and an air level in the thoracic aorta and the left ventricle (Fig. 1c and 1d). On transferring the patient back to the supine position, he experienced a brief period of absence with conjugate eye deviation, but breathing and circulation were undisturbed. Subsequently, the patient was transferred to an intensive care unit for surveillance, where the symptoms rapidly regressed without any sequelae. A magnetic resonance imaging (MRI) scan of the cerebrum conducted 1 h later revealed no significant abnormalities next to the known cerebral metastases.

### Further patient follow-up

Histopathological analysis of the retrieved specimen later revealed an epidermal growth factor receptor (EGFR) negative adenocarcinoma. A reinduction chemotherapy with carboplatin and pemetrexed was initiated, lasting to September 2015. Since then, two more lines of chemotherapy have been applied on progression of cerebral and pulmonary lesions, respectively. At the moment, the patient is undergoing immunotherapy with pembrolizumab, whereas a regression of several pulmonary lesions and stable disease in abdominal and cerebral metastases has been reported in the latest restaging scans in September 2017.

## Discussion

### CT-guided lung biopsy: technique and diagnostic performance

A differentiation can be made between two major approaches to PCNB, namely fine-needle aspiration (FNA) and core (cutting) needle biopsy (CNB). The FNA approach uses a hollow needle with a diameter smaller than 22 gauge (G) for aspiration and usually allows only cytological analyses of the specimen, while CNB (20 G or larger) can excise tissue samples of a size that enables full histological examination [1–3]. Especially regarding lung carcinoma, CNB reportedly has a superior diagnostic yield compared to FNA, providing material sufficient for full molecular testing in >90% of cases [1, 6, 7]. As target therapy in lung cancer is rapidly evolving, an increasing future need for minimally invasive specification of pulmonary lesions can be anticipated. Also, the emerging field of lung cancer screening is likely to cause a rising demand for transthoracic lung biopsies, especially when up-front surgical resection is considered inappropriate or impossible [4, 8].

### Frequent complications in CT-guided lung biopsy

A recent meta-analysis, comprising 8133 CNB and 4620 FNA procedures, reported overall complication rates of 38.8% with 5.7% severe complications for CNB, versus 24% and 4.4% for FNA, respectively. The overall complication rate was significantly higher in CNB; however, the occurrence of severe complications including pneumothorax requiring intervention, hemothorax, air embolism, needle tract seeding, and death did not differ significantly between the groups. The most frequent complication for both CNB and FNA was pneumothorax, occurring in 25.3% and 18.8% of cases, respectively. An intervention was required in 5.6% and 4.3%, respectively. The reported overall rate of pulmonary hemorrhage after CNB and FNA was 18% and 6.4%, respectively, with hemoptysis in 4.1 and 1.7% [4].

Other studies comprised in the ACR (American College of Radiology)/SIR (Society of Interventional Radiology) quality improvement guidelines for percutaneous needle biopsy, reported a similar pneumothorax rate of 12–45%, requiring chest tube placement in 2–15% [3]. A review of 102 cases using CT-guided Tru-Cut biopsy reported a pneumothorax rate of 15.7% and a chest tube placement rate of 8.8% [5]. The overall complication rate seems to increase with the needle diameter, whereas 22 G may constitute the threshold between higher and lower risk [4]. In addition, a smaller lesion diameter and more traversed lung parenchyma have been identified as predictors for a higher complication rate. Other prognostic factors proposed include patient age, the presence of emphysema and a larger lesion depth [1–5]. Various strategies to minimize the complication risk especially for pneumothorax have been proposed, including preferentially prone patient position, sedation and the use of CT fluoroscopy. Also, up-front puncture site-down positioning or immediate rolling-over to this position after biopsy have been discussed [1, 4, 9, 10].

### Rare but underestimated: systemic air embolism

Systemic air embolism is generally believed to be a very rare complication, with a reported incidence of 0.04–0.07% in large registries [3, 11–13]; however, this potentially fatal adverse event may be frequently overlooked and thus underreported, as implied by Freund et al., who analysed control CT sequences recorded directly after biopsy. Using a standardized algorithm, systemic air embolism could be retrospectively detected in as many as 3.8% of cases, although only 0.49% were symptomatic and 0.16% were fatal [14].

The pathogenesis of air embolism may be complex; however, what several explanatory models have in common is the access of air into the pulmonary vasculature following a pressure gradient. This is usually created by an additional factor, leading to either higher intrabronchial pressure, or to decreased endovascular pressure [13–15]. In line with these theoretical physical approaches, risk factors for systemic air embolism identified in various studies include a lesion level above the left atrium, prone patient position and positive pressure ventilation. Other mentioned procedure-related aspects possibly favoring systemic air embolism are the use of a coaxial needle, larger biopsy needle diameter and the depth of the needle within the lesion [12, 14, 15]. Cavitary lesions, the presence of vasculitis or mycosis, coughing or a Valsalva manoeuvre during the procedure are frequently named as patient-sided risk factors [12–15].

The clinical presentation of systemic air embolism depends on which organ is impaired by the influx of air bubbles. This includes cardiac failure, arrhythmia and myocardial infarction for heart manifestations due to coronary embolism or intracardiac air-lock as well as neurological abnormalities when cerebral arteries are affected [14, 16]; however, as only 26% of the radiologically proven air embolism events have been reported to be clinically evident, [14] it is likely that a high number of patients with systemic air embolism remain asymptomatic.

In cases of suspected or proven systemic air embolism, the literature suggests immediate prevention of further entry of air, hemodynamic support as necessary up to cardiopulmonary resuscitation, application of 100% oxygen and administration of heparin. If possible, the patient should be placed in a left lateral decubitus position to prevent air-lock in cases of cardiac failure or in Trendelenburg position in cases of neurological symptoms [1, 14–17]. Additionally, in selected cases, aspiration of air from the right atrium can be considered, as well as hyperbaric oxygen therapy [16, 17].

### Minimizing the risk of systemic air embolism

Among the numerous prevention strategies against systemic air embolism that have been suggested, many remain controversial as they are partly inconsistent, and a prospective evaluation is difficult, given the rare incidence. For example, prone patient position is named a risk factor [14]; however, this is by far the most usual position the procedure is conducted in [12, 15]. Turning the patient immediately after biopsy may reportedly result in lower pneumothorax rates [4]. Conversely, considering anatomical conditions, this manoeuvre could cause acute embolization of air bubbles trapped in the aortic arch or in the left heart, which probably happened in the aforementioned patient case [15]. Thus, at least concerning the risk of systemic air embolism, the control scan following biopsy should be conducted in an unaltered position [15]. When a coaxial needle is used, the inner cannula should be blocked as fast as possible to prevent the influx of air, e. g. by using a hemostatic valve assembled to the biopsy needle [1, 15]. Other more general procedural considerations for preventing air embolism include avoiding pleural fissures, emphysematous areas and passing through a long length of lung tissue [14, 15].

## Conclusion

Percutaneous CT-guided transthoracic lung biopsies have become everyday clinical practice. In times of target therapy, there is a growing demand for re-biopsies as well as for high-quality and quantity tissue samples enabling extensive panels of molecular pathology and mutation testing. In this context, one must not forget that especially core biopsies bear a substantial risk of complications. As our case report shows, also rare and unexpected incidents such as systemic air embolism following lung biopsy can quickly put patients’ lives at risk. Given the high complication rate, clinicians need to be aware of such potentially fatal complications and should be prepared to provide effective emergency treatment at any time. To increase patient safety in PCNB, there is an urgent need for a prospective evaluation of risk factor management strategies. More evidence-based knowledge of such patient, device and procedure-related aspects would enable a risk stratification before the intervention and could minimize the hazard the patient needs to be exposed to.

## References

[1] Winokur RS, Pua BB, Sullivan BW, Madoff DC (2013). Percutaneous lung biopsy: technique, efficacy, and complications. Semin Intervent Radiol.

[2] Manhire A, Charig M, Clelland C, BTS, - (2003). Guidelines for radiologically guided lung biopsy. Thorax.

[3] Gupta S, Wallace MJ, Cardella JF, Kundu S, Miller DL, Rose SC, Society of Interventional Radiology Standards of Practice Committee (2010). Quality improvement guidelines for percutaneous needle biopsy. J Vasc Interv Radiol.

[4] Heerink WJ, de Bock GH, de Jonge GJ, Groen HJM, Vliegenthart R, Qudkerk M (2017). Complication rates of CT-guided transthoracic lung biopsy: meta-analysis. Eur Radiol.

[5] Beşir FH, Altın R, Kart L (2011). The results of computed tomography guided tru-cut transthoracic biopsy: complications and related risk factors. Wien Klin Wochenschr.

[6] Beslic S, Zukic F, Milisic S (2012). Percutaneous transthoracic CT guided biopsies of lung lesions; fine needle aspiration biopsy versus core biopsy. Radiol Oncol.

[7] Tian P, Wang Y, Li L, Zhou Y, Luo W, Li W (2017). CT-guided transthoracic core needle biopsy for small pulmonary lesions: diagnostic performance and adequacy for molecular testing. J Thorac Dis.

[8] Vansteenkiste J, Crinò L, Dooms C, Panel Members (2014). 2nd ESMO consensus conference on lung cancer: early-stage non-small-cell lung cancer consensus on diagnosis, treatment and follow-up. Ann Oncol.

[9] Prosch H, Stadler A, Schilling M (2012). CT fluoroscopy-guided vs. multislice CT biopsy mode-guided lung biopsies: accuracy, complications and radiation dose. Eur J Radiol.

[10] Kinoshita F, Kato T, Sugiura K (2006). CT-guided transthoracic needle biopsy using a puncture site-down positioning technique. AJR Am. J. Roentgenol..

[11] Tomiyama N, Yasuhara Y, Nakajima Y (2006). CT-guided needle biopsy of lung lesions: a survey of severe complication based on 9783 biopsies in Japan. Eur J Radiol.

[12] Ishii H, Hiraki T, Gobara H (2014). Risk factors for systemic air embolism as a complication of percutaneous CT-guided lung biopsy: multicenter case-control study. Cardiovasc Intervent Radiol.

[13] Hiraki T, Fujiwara H, Sakurai J (2007). Nonfatal systemic air embolism complicating percutaneous CT-guided transthoracic needle biopsy: four cases from a single institution. Chest.

[14] Freund MC, Petersen J, Goder KC, Bunse T, Wiedermann F, Glodny B (2012). Systemic air embolism during percutaneous core needle biopsy of the lung: frequency and risk factors. BMC Pulm. Med..

[15] Rott G, Boecker F (2014). Influenceable and avoidable risk factors for systemic air embolism due to percutaneous CT-guided lung biopsy: patient positioning and coaxial biopsy technique-case report, systematic literature review, and a technical note. Radiol Res Pract.

[16] Mirski MA, Lele AV, Fitzsimmons L, Toung TJ (2007). Diagnosis and treatment of vascular air embolism. Anesthesiology.

[17] Bou-Assaly W, Pernicano P, Hoeffner E (2010). Systemic air embolism after transthoracic lung biopsy: a case report and review of literature. World J Radiol.

